# Insensitivity to Flaws Leads to Damage Tolerance in Brittle Architected Meta-Materials

**DOI:** 10.1038/srep20570

**Published:** 2016-02-03

**Authors:** L. C. Montemayor, W. H. Wong, Y.-W. Zhang, J. R. Greer

**Affiliations:** 1 California Institute of Technology, Pasadena, CA, USA; 2 Institute of High Performance Computing, (A*STAR), 1 Fusionopolis Way, #16-16 Connexis, Singapore 138632

## Abstract

Cellular solids are instrumental in creating lightweight, strong, and damage-tolerant engineering materials. By extending feature size down to the nanoscale, we simultaneously exploit the architecture and material size effects to substantially enhance structural integrity of architected meta-materials. We discovered that hollow-tube alumina nanolattices with 3D kagome geometry that contained pre-fabricated flaws always failed at the same load as the pristine specimens when the ratio of notch length *(a)* to sample width *(w)* is no greater than 1/3, with no correlation between failure occurring at or away from the notch. Samples with *(a/w)* > 0.3, and notch length-to-unit cell size ratios of *(a/l)* > 5.2, failed at a lower peak loads because of the higher sample compliance when fewer unit cells span the intact region. Finite element simulations show that the failure is governed by purely tensile loading for *(a/w)* < 0.3 for the same *(a/l)*; bending begins to play a significant role in failure as *(a/w)* increases. This experimental and computational work demonstrates that the discrete-continuum duality of architected structural meta-materials may give rise to their damage tolerance and insensitivity of failure to the presence of flaws even when made entirely of intrinsically brittle materials.

Bulk ceramics are highly sensitive to flaws and fail catastrophically upon applied loads, most commonly at the small internal flaws like cracks, voids, and inclusions^1^,^2^,^3^. Fiber-reinforced ceramic-matrix composites (CMCs) have been developed to reduce their sensitivity to flaws while capitalizing on the high strength of ceramics^4^,^5^,^6^,^7^. These CMCs utilize deformation of the matrix and/or fibers to delocalize strain near stress concentrators, such as holes or notches, which leads to flaw insensitive behavior^4^,^5^,^7^. Insensitivity to notches has been reported for silicon carbide/calcium aluminosilicate CMCs for ratios of notch to sample size of 0.2 < *(a*_*0*_*/b)* < 0.8, where *a*_*0*_ is the notch size and *2b* is the sample width^5^,^7^.

It has also been postulated that reducing sample dimensions of brittle materials can give rise to flaw insensitivity and to attain near-theoretical strength^8^. Gao *et al.*’s theoretical work demonstrated that a thin plate with a penny shaped notch exhibits the fracture strength of a perfect crystal when the plate thickness falls below a critical length scale, which is a function of surface energy, elastic modulus, and ideal material strength^8^. Nano-fracture experiments and computations on brittle nanocrystalline platinum nanopillars, with diameters of 100 nm and a grain size of 6 nm, revealed that their failure strength remained equivalent to the ultimate tensile strength even in the presence of pre-fabricated flaws and that failure location was uncorrelated to the presence of the flaws^9^. This emergence of flaw insensitivity in nano-structures was attributed to their failure at the “weakest-link,” be it at an internal, microstructural stress concentration like a grain boundary triple junction or at an external flaw, with failure mechanism representing the intrinsic material strength. In the absence of a discrete material microstructure, it has been reported that 75 nm-diameter metallic glass nanopillars containing external notches always failed at those locations at lower peak loads than their un-notched counterparts^10^.

These examples demonstrate that failure tolerance of some materials to flaws cannot be solely attributed to the length scale; it stems from the complex interplay between the internal microstructural energy landscape within the material and the external sample dimensions and geometry^10^. Several studies have demonstrated that incorporating architecture in material design enables proliferating lucrative material size effects that emerge at the nano-scale onto macro-scale architected meta-materials; for example *smaller is stronger/weaker* for metals, *smaller is ductile* for brittle metallic glasses, and *smaller is tougher* for ceramics^11^. The periodic arrangements of small-scale ordered cellular solids, such as nano- or meso-lattices, span length scales ranging from hundreds of microns to tens of nanometers and facilitate the attainment of novel mechanical properties under compression, like recoverability and enhanced specific strength compared to bulk, and these properties arise as a result of structural and material size effects^12^,^13^,^14^. Existing cellular solids theories predict that mechanical behavior is determined by the deformation mechanism of the lattice, which is either by bending or stretching and is a function of the nodal connectivity, and the constituent material properties^15^,^16^,^17^. A bending dominated structure is predicted to have lower strength and stiffness when compared to a stretching-dominated structure^15^,^16^,^17^,^18^. In the case of architected bulk materials, another aspect of microstructure arises in the dimensions of not only the grains of the constituent material but also in the size of the unit cells. Fracture experiments on macro-scale cellular solids have been explored in literature; tensile properties of nanolattices – with or without pre-fabricated defects - are currently unknown^16^,^19^,^20^,^21^,^22^,^23^,^24^,^25^,^26^.

We explore tensile failure of 3-dimensional hollow alumina kagome nanolattices and demonstrate that they exhibit flaw tolerance in terms of strength and failure location, which we attribute to the presence of a discrete structure at the micron and sub-micron lengths scales within a continuum-like material.

## Kagome Tension Sample Fabrication

**Figure 1:**
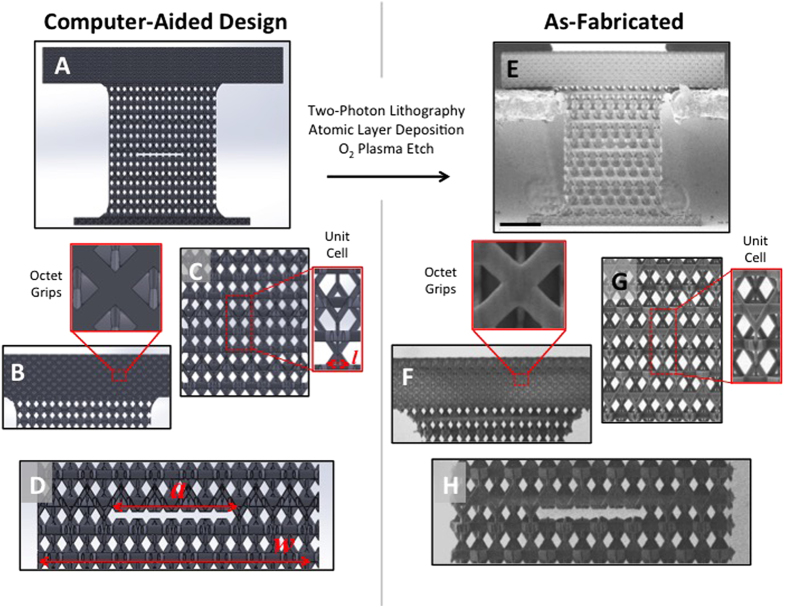
Representative notched nanolattice tensile specimens, designed in Solidworks (**A**) and as-fabricated samples (**E**). The fabricated and designed unit cells, whose size denoted as *l*, are shown in (**C**,**G**), respectively. The grip used to apply uniaxial tension to the samples is an octet-truss lattice, shown in (**B**,**F**). The notch is shown in (**D**,**H**) and is denoted by the variable *a* while the sample width is denoted by *w*. Scale bar (**E**) denotes 25 μm.

We performed uniaxial tension experiments on hollow alumina (Al_2_O_3_) nanolattices with and without through-thickness notches. Figure 1 shows the CAD design and SEM images of an as-fabricated dog-bone-shaped hollow alumina kagome nanolattice thin plate embedded in an octet-truss lattice head; the kagome lattice had a unit cell size of *l* = 3.85 ± 0.16 μm, the octet-truss head had a unit cell size of *l* ~ 4.5 μm, shown in Fig. 1(B,F). The kagome lattice was created by tessellating pairs of stacked tetrahedra in three dimensions in a hexagonal tessellation pattern, which gives rise to an A-B-C stacking pattern. The kagome unit cell shown in Fig. 1(C,G) contains a single complete set of A-B-C stacked tetrahedra. The tension samples were fabricated on a silicon wafer using a polymer scaffold created via two-photon lithography direct laser writing process and were subsequently coated with 50 nm of Al_2_O_3_ using atomic layer deposition (ALD) at 150 °C to make a composite Al_2_O_3_/polymer structure. The octet-truss head had a width of *w* = 139.8 ± 1.1 μm, a height of *h* = 19.2 ± 0.2 μm, and a thickness of *t* = 24.5 ± 0.3 μm, with the averages representing 5 samples. The Al_2_O_3_/polymer kagome test section initially had a width of 12 unit cells, a height of 6 unit cells, and a thickness of 2  unit cells but the width was reduced at a later step in the fabrication process to accommodate the experimental set-up. The samples had an octet-truss lattice at both the top and bottom of the kagome test section, shown in Fig. 1(A,E); a kagome A-B-C unit cell layer was embedded in octet-truss head and ~5 μm of the kagome test section was embedded in the bottom octet-truss lattice at the interface between the sample and the silicon wafer. The kagome lattice was embedded in the octet-truss lattices at the top and bottom of the test sections to avoid delamination at the interfaces between the kagome test section and the octet-truss head/substrate under tensile loading.

Samples were shaped into dog-bone tensile geometries using a focused ion beam (FIB), at a current of 7 nA, to have a curved edge at the kagome/octet-truss interfaces that served to reduce stress concentrations (Fig. 1(B,F)). The plate-like kagome Al_2_O_3_/polymer test section had a final width of *w* = 66.3 ± 5.2 μm, a height of *h* = 81.9 ± 0.3 μm, and a thickness of *t* = 15.4 ± 0.5 μm; the width and height averages were calculated using all samples presented in this work (n = 18) and the average thickness was determined by averaging the thickness of 10 representative samples after the deformation. The FIB was also used to pattern the notches (Fig. 1(D,H)). We tested samples with notch lengths, *a,* ranging from 0 to 0 − 34.94 μm, and *(a/w)* varying from 0–0.54, where *w* is the width of the test section; the unit cell size was kept constant for all samples and notch length-to-unit cell size ratio, *(a/l)*, was varied from 0–9.1. Once the samples were shaped into their final geometry, the internal polymer was removed using O_2_ plasma (16–18 hours at 100 W) and the samples were visually inspected in the scanning electron microscope (SEM) to non-destructively determine the amount of polymer remaining in the sample; the amount of polymer removed was verified to be approximately constant across all samples using this technique (see Supplementary Info). When the Al_2_O_3_ kagome nanolattices were fully hollowed out, the octet-truss head remained a composite, containing the Al_2_O_3_ layer and the internal polymer. Additional details on the fabrication process can be found in refs 12, 13, 14, 27 and 28.

We chose the 3D kagome geometry because of its high predicted fracture toughness in 2D, which stems from elastic blunting near the crack tip^16^,^24^. The hollow kagome nanolattices in this work are designed to have a stiffness of *E* = 45 MPa and a relative density of 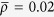, calculated using a Solidworks model of the kagome unit cell, shown in Fig. 1, and the measured sample dimensions from SEM images. We chose the denser Al_2_O_3_/polymer octet-truss lattice as the sample head to stiffen this section relative to the hollow kagome test section^16^,^18^. The composite Al_2_O_3_/polymer head has a 760x higher stiffness of *E* = 3.90 GPa and a relative density of 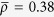 (see Supplementary Info for details). We find that this difference in stiffnesses was sufficient to perform the tensile loading with minimized deformation of the octet-truss head.

## *In-Situ* Uniaxial Experiments and Simulations

The as-fabricated kagome dog-bone specimens were subjected to displacement rate-controlled uniaxial straining in an *in-situ* nanomechanical instrument, InSEM (Nanomechanics, Inc.), at a quasi-static strain rate of 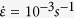. Contact with the samples was made via a tension grip at the bottom faces of the octet-truss head on either side of the kagome lattice; the tension grip was milled in the head of a 0.8 mm stainless steel screw using electrical discharge machining (EDM), as shown in Fig. 1E. Load-displacement data, as well as real-time video of the deformation, was captured during each uniaxial tension experiment. The displacement of the gauge section was calculated using the observed length change, Δ*l,* in the deformation video; uniaxial strain was defined as *ε* *=* Δ*l/l*, with the original length *l* measured in SEM prior to the experiment. The stress at failure was defined as *σ* = *F/A*, where *A* is the overall cross-sectional area of the sample and *F* is the measured force at failure. The slope of the unloading curve is not a reliable measure of the energy release-rate of the Al_2_O_3_ kagome nanolattice since the slope is an artifact of the InSEM controller.

Finite Element (FE) simulations of the as-designed notched and un-notched hollow kagome lattices were performed to assess the ability of continuum-based models to predict deformation of architected meta-materials. The samples in FE models were created from the SolidWorks-constructed geometries and accounted for the interface between the octet-truss sample head and the InSEM grips. Three-dimensional 3-noded triangular shell elements with reduced-integration were employed. The material properties of Al_2_O_3_ for the FE analyses were obtained from bulge experiments of equivalently deposited thin films of ALD Al_2_O_3_, with the modulus, E, ranging from 164–165 GPa and the ultimate tensile strength, σ_UTS_, in the range of 1.57–2.56 GPa; for the simulations in this work, the input modulus and UTS were taken to be 165 GPa and 1.57 GPa, respectively^29^. A linear post-cracking stress-strain relationship was incorporated in the simulations to represent the brittle behavior of Al_2_O_3_. Displacement-controlled boundary conditions were applied to the octet-truss sample head in locations closely resembling the experimental setup. For numerical efficiency, explicit dynamics procedure was adopted to model the quasi-static applied uniaxial tension loading. To ensure a quasi-static response, the energy balance of the modeling system was constantly monitored such that the kinetic energy of the system was negligible compared to its internal energy and external work. Computations were performed within the finite strain setting using the general-purpose finite element program ABAQUS/Explicit Version 6.13.2. To model and reflect a quasi-static solution, the kinetic energy of the deforming kagome lattice was monitored and enforced to not exceed 1% of its internal energy throughout the majority of the quasi-static analysis. The studied *(a/w)* ratios were: 0, 0.11, 0.23, 0.35, 0.47 and 0.54. A constant wall thickness of 50 nm across all elements was assumed for all simulations. The global stress and strain of the FE kagome test section was calculated using the same methodology as in experiments.

## Results

**Figure 2:**
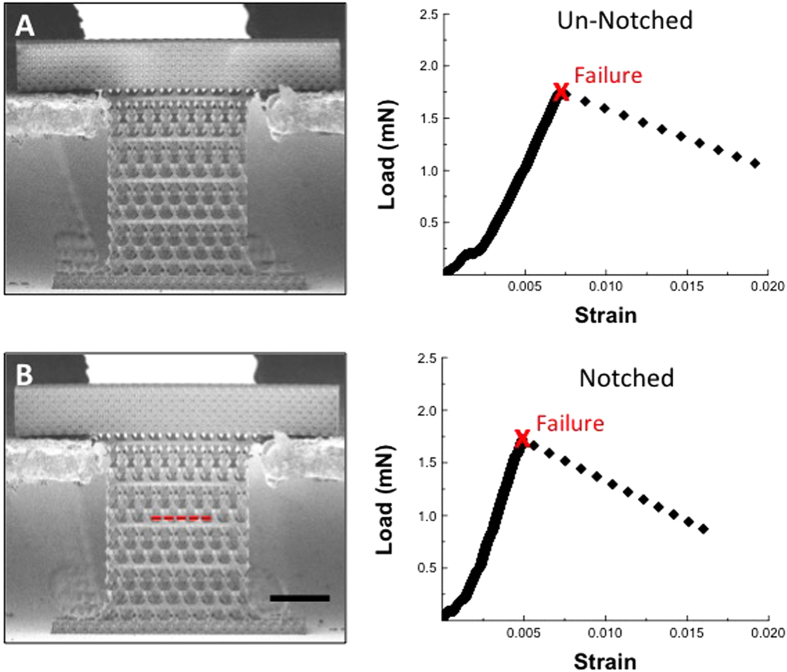
Representative load-displacement curve for un-notched (**A**) and notched (**B**) kagome nanolattices (4 μm unit cell, 50 nm Al2O3) in uniaxial tension. The dimensions of the notched and un-notched samples shown are identical with the exception a notch, which spans 1/3 of the sample width and is denoted by a red dashed red line. The red “X” denotes brittle, catastrophic failure and all data collected after this point is an artifact of the testing instrument and not representative of the measured load on the sample Scale bar denotes 25 μm.

**Figure 3:**
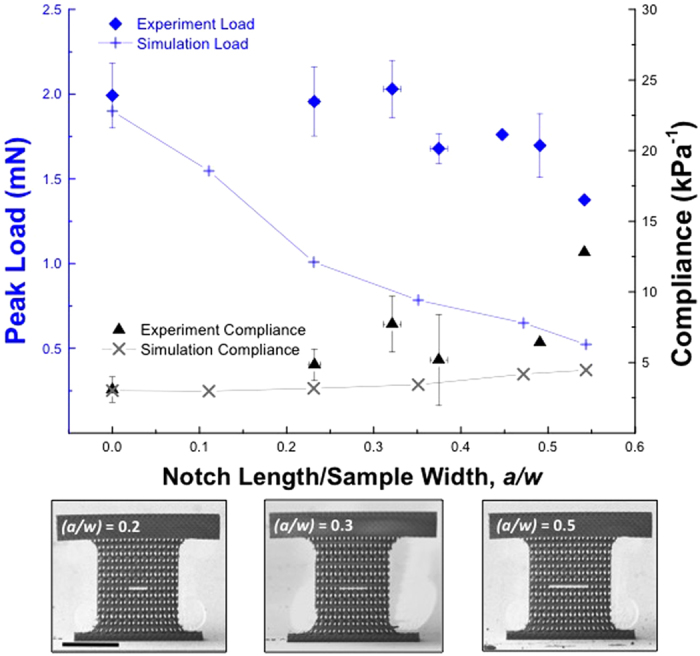
Comparison of finite element and experimental data for 4 μm kagome lattices in uniaxial tension. Scale bar denotes 50 μm in all images. The error bars in the experimental data were calculated using the standard deviation of the measured load and notch/sample dimensions; substantial errors were likely caused by the variations in the compliance of the sample head.

Figure 2 shows SEM images and representative load vs. strain-to-failure data for the as-fabricated and notched geometries and reveals that all samples failed instantaneously and catastrophically, as expected for a brittle ceramic. Three un-notched samples (*a/w* = 0) had a *F*_*peak*_ = 2.00 ± 0.19 mN and a strain at failure of *ε*_*failure*_ = 0.006 ± 0.001; the results for various notch sizes are tabulated in the Supplementary Information. Figure 3 shows peak-load-at-failure data for samples with notch-to-width ratios, (*a/w*), spanning from 0 to 0.54. Samples with *(a/w)* between 0 and 0.32 had a relatively constant peak-load-at-failure of ~ 2 mN, which is equivalent to the peak load for the un-notched samples. The peak load decreased by 32% as *(a/w)* increased from 0.32–0.54, likely caused by the 4.2x higher compliance in the widest-notched samples when compared to un-notched samples, also shown in Fig. 3. The experimentally obtained peak load remained nearly constant over the *(a/w)* range of 0–0.32; the simulations show a monotonic reduction in the peak load with increasing *(a/w)*, decreasing by 59% as *(a/w)* widens from 0–0.35. The simulations also show a 1.5x increase in compliance as *(a/w)* increases from 0–0.54, slightly lower than that observed experimentally.

The sample compliance was calculated as C = *ε*/σ, where σ is the applied load divided by the full cross-sectional area of the gauge section, *A*, for both experiments and simulations. The experimentally measured compliance is the combined compliance of the sample/nanoindenter system and may not serve as a reliable measure of the absolute compliance of the nanolattices; the calculated compliance serves to compare the relative changes in sample compliance with increasing ratios of *(a/w)*.

## Discussion

**Figure 4:**
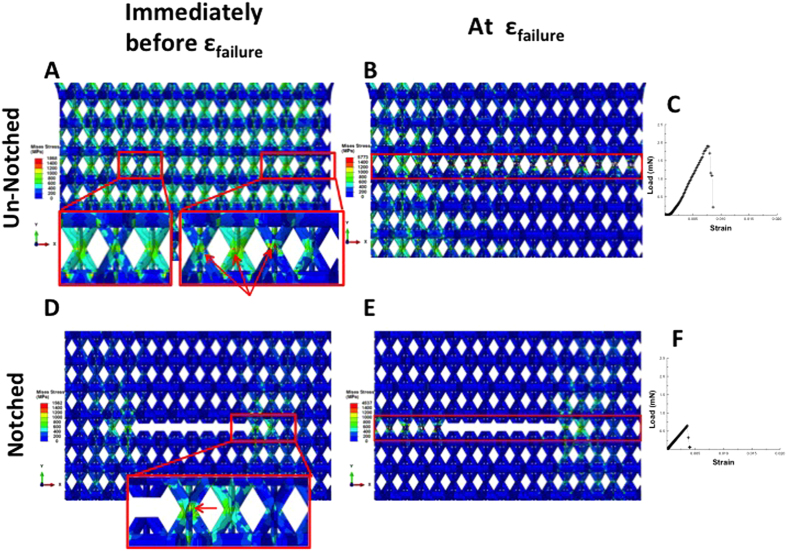
Finite element simulations show that the highest local Von Mises stress concentrations within the nanolattice occur at the nodes for both the notched and un-notched specimens. Red boxes denote the plane where failure occurs in the samples (**B**,**E**) and red arrows denote the highest visible surface stress concentrations immediately before failure (**A**,**D**).

All samples in this work failed catastrophically for all *(a/w),* with failure always occurring along a plane of nodes between the tetrahedral pairs forming the kagome lattice. FE simulations revealed that the highest local Von Mises stresses occur at the nodes, along the “planes” where the tetrahedra connect; Fig. 4 shows these calculations for representative un-notched and notched (*a/w* = 0.3) samples and reveals that the nodes serve as the weakest links when the nanolattice is tensed. This is not surprising, as it has been previously demonstrated that the nodes in similarly-made Al_2_O_3_ nanolattices were the weakest links and served as failure initiation locations in compression^12^,^14^. We discovered that the as-fabricated, un-notched samples have equivalent local stresses in the nodes located in sample-interior and at the edge of the sample and that the notched samples have the highest local stress concentrations at the notch roots, with minimal local stress at the sample edge immediately prior to failure. Failure in each sample initiated at the node(s) that had a missing neighbor, whose detached side was not constrained by the neighboring unit cells in the lattice. Failure in the notched samples always initiated at the notch root where the adjacent unit cell had one constrained and one un-constrained boundary. All samples in this work had one edge containing unit cells that were fully disconnected from their vertical neighbors while the other edge had unit cells with minimal intact vertical connections along the sample edge; this is a result of the lattice unit cell size and the sample width required to fit the tension grips. Failure in all un-notched samples initiated at the edge with completely disconnected unit cells, which is similar to local geometry at the notch edge where the unit cells have are disconnected from the rest of the lattice on one side. After incipient failure in a single edge node, the crack propagates instantaneously and causes catastrophic failure of the entire structure; the applied force required to fail the first node in uniaxial tension corresponds to the peak load at failure for a nanolattice.

While continuum-based classical mechanics theory predicts that the peak load will decrease at higher *(a/w)*; the nanolattices here exhibit a nearly constant peak load of ~2 mN for *(a/w)* < 0.38, a value equivalent to that of the un-notched material^1^,^2^,^30^,^31^. In monolithic ceramic materials with the same geometry, the stress concentration at a notch is highest because the external notch is significantly larger than the size of the internal microstructural flaws^1^,^2^,^3^. Our simulations indicate that in a nanolattice, the stress concentrations at the nodes (internal) and at the notch (external) have similar magnitude because they both arise from the discrete nature of the periodic unit cells. These findings are consistent with tension experiments on notched nanocrystalline Pt nano-tension specimens, which show that the internal stress concentrations at triple junctions and at grain boundaries are comparable to the stress concentrations at the external notches^9^. The constancy of the peak load at failure arises from the global deformation in all samples being governed by single-node failure in uniaxial tension (provided the sample compliances are comparable). As *(a/w)* increases to above ~ 0.38, the observed peak load decreases, likely because the remaining intact unit cells are less constrained and experience bending moments. Fleck *et al.* used FE simulations to show that 2D kagome lattices with solid beams exhibit elastic blunting at the crack tip that results from the stretching-periodic bending geometry, where the lattice bars deform by bending and stretching in the vicinity of the crack. It was also predicted that the radius of the crack blunting region can extend to lengths of up to 20 unit cells for lattices with 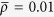
32. The lateral displacements calculated for each sample here did not reveal any elastic blunting, likely because the kagome nanolattices in this work contain hollow, non-slender tubes while the work of Fleck *et al.* assumes solid, slender beams^32^.

**Figure 5:**
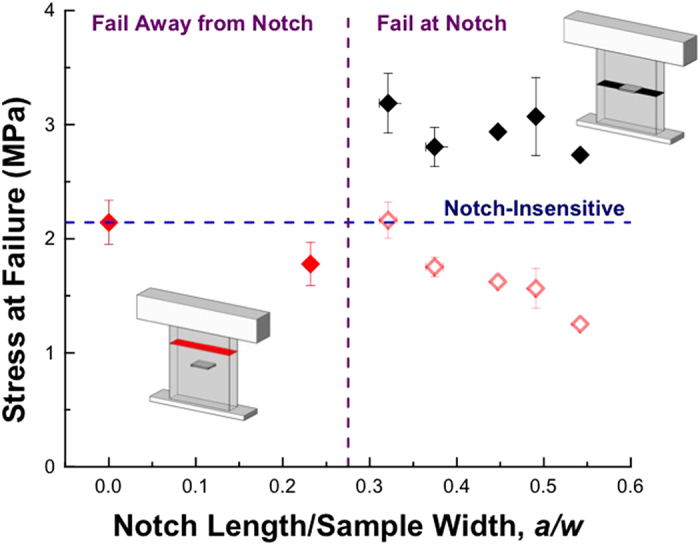
Stress at failure vs. the notch length to unit cell size ratio. Solid symbols denote samples where the area used to normalize the load corresponds to the location of failure, whether at or away from the notch. Red symbols denote the peak load normalized by the total cross-sectional area of the tension specimen; black symbols denote the peak load normalized by the attached area at the notch plane. The blue dashed lined denotes the trend expected for a notch-insensitive material. For samples failing at the notch and normalized by the intact area at the notch plane, the load is roughly constant and higher than the un-notched samples due to the reduced area at the notch plane.

All nanolattices with *(a/w)* ≤ 0.23 failed away from the notch with the peak load remaining constant at *F*_*peak*_ ~ 2mN; nanolattices with wider notches failed along the plane of the notch, orthogonal to the loading direction, and *F*_*peak*_ decreased with a concomitant increase in compliance. Figure 5 summarizes the stress at failure as a function of relative notch size and outlines failure location property space. Failure stress was determined by dividing the peak load, *F*_*peak*_, by the cross-sectional area, A, which was calculated using two different criteria: (1) the intact area at the plane of failure (black diamonds)^4^,^5^ and (2) the total cross-sectional area of the gauge section (red diamonds). Using the first approach, we observed a roughly constant stress at failure of ~3MPa for *(a/w)* > 0.3, a plateau that is consistent with literature^4^,^5^. Using the intact area at the notch plane to calculate the stress at failure results in its increase by a factor of 1.8 when *(a/w)* increased from 0.23–0.32, which corresponds to a transition in failure location. A lower stress at failure of 2.73 MPa for the widest-spanning notch, *(a/w)* = 0.54, is likely a result of substantial compliance, 12.8 kPa^−1^, of this sample. To determine the stress at failure for *(a/w)* ≥ 0.54, samples with more unit cells across the width must be fabricated; the fabrication method used in this work poses limitations on creating significantly larger, structurally robust samples of the same geometry. When the stress at failure is calculated using the full cross-sectional area, a parameter space that is insensitive to flaws exists for *(a/w)* < 0.38 where the stress at failure is ~2.14 MPa. A reduction in stress at failure as *(a/w)* increases from 0.38–0.54, calculated using this method, stems from the higher sample compliance for *(a/w)* > 0.38, which wouldn’t occur for larger samples with more unit cells spanning the un-notched region. This analysis demonstrates that hollow Al_2_O_3_ nanolattices are insensitive to the presence of external flaws for pre-fabricated defects spanning up to 30% of the sample width. We postulate that this flaw insensitivity arises from the discrete nature of the nanolattice at the micron scale such that the failure strength for notched and un-notched samples is governed by the failure strength of individual junctions between the unit cells subjected to uniaxial tension.

**Figure 6:**
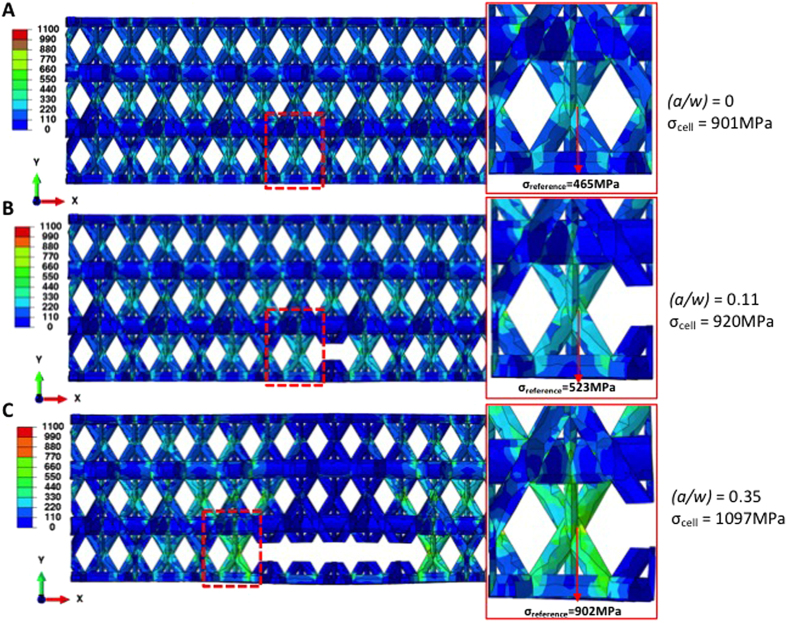
Contour plots of von Mises stress distributions in kagome lattice at a nominal strain of ε_y_ = 0.003 for *(a/w)* = 0 (**A**), 0.11 (**B**) and 0.35 (**C**). The distortions shown are magnified by 5 times to enhance visualization. Insets are the close-up views of the unit cell at the notch root. σ_reference_ denotes the von Mises stress at the same location on the junction node in the unit cell, and σ_cell_ refers to the maximum von Mises stress in the unit cell.

Figure 4 shows representative load-strain data for the notched and un-notched samples generated using FE simulations, which predict a 2.5-times lower peak load for the notched sample compared to the un-notched sample. The model predicted the same peak load as experimentally observed for the un-notched samples and severely under-predicted it for the notched sample with *(a/w)* = 0.32 (Figs 3 and 4). We examined the sample region that contains the notch and the unit cell at the notch root, with the same region in the un-notched sample chosen as a reference for comparison. Figure 6 displays the von Mises stress distributions and associated distortions in the samples with *(a/w)* = 0, 0.11 and 0.35 at an applied nominal strain of ε_y_ = 0.003, which is well within the elastic regime of the deformation. We denote σ_reference_ as the von Mises stress at the junction node in the notch root unit cell, pointed to by the arrows in the zoomed view, and σ_cell_ as the maximum von Mises stress in the unit cell. Using the un-notched lattice, with (*a/w* = 0), as a reference, simulations reveal that a short notch (*a/w* = 0.11) leads to the stress distributions and the associated distortions being comparable to those in the un-notched lattice. When the notch is lengthened to *(a/w)* = 0.35, the stresses in the unit cells above and below the notch become significantly lower, which generates a large stress concentration at the notch root. For the short notches, the relative change in σ_reference_ is 13% and that in σ_cell_ is 2%; those for long notches are 94% and 22%, respectively. Figure 6B also shows that for the short-notch case, the distortions are marginal and similar to those in the un-notched samples, which corroborate the observed notch-insensitivity in experiments. For longer notches, the distortions become significant, with a shift in local stresses towards the notch roots. The insets in Fig. 6B display a zoomed-in view of the notch root and show that for samples with a short notch, the node at the notch root experiences close-to-uniaxial tensile loading mode, which is similar to that in the samples without the notch (Fig. 6A). In samples with long notches (Fig. 6C), the node adjacent to the notch is subject to bending in addition to tension. Failure in long-notched samples initiates at the notch root node; the transition in stress state from tensile-only in the un- and short-notched samples to tensile-and-bending in the long-notched ones may cause different failure modes, which define the notch-insensitivity and notch-sensitivity regimes.

The observed discrepancies between the computations and experimental results shown in Fig. 3 can be attributed to several factors. First, the compliance of the experimental setup cannot be accurately accounted for in the numerical simulations, which leads to a lower calculated peak loads. Secondly, the beam wall thickness and diameter, as well as the hollowness of the beams in the gauge section and Al_2_O_3_/polymer composite in the sample head, are assumed to be perfectly uniform in the simulations, which is an idealization of the as-fabricated structure. Additional calculations indicate that variations in tube wall thickness lead to a higher predicted peak load because of the greater load-bearing capacity of the trusses (See Supplementary Information). During sample preparation, the internal polymer of the sample head may be partially exposed to oxygen plasma near the kagome/octet interface, which would lead to variations in the stiffness of the sample head if the internal polymer inside is partially etched (See Supplementary Info). Our simulations revealed that the stress state at the notch root node changed from uniaxial tension to a combination of tension and bending with increasing notch length, which may render the maximum tensile stress-based failure criterion invalid. Several strategies are being pursued to improve the current numerical model, which include incorporating an inhomogeneous distribution of beam wall thicknesses by considering a statistical distribution of thicknesses based on experimental measurements, developing a better understanding of constituent material properties, and designing a more suitable failure criterion.

## Summary/Conclusion

We fabricated 3-dimensional dog-bone shaped, hollow-beam alumina nanolattices with kagome architecture, with and without through-notches that span up to 30% of the sample width, and performed uniaxial tension experiments on them. These alumina nanolattices exhibit a nearly constant load-at-failure for notch length-to-sample width ratios of *(a/w)* < 0.38, which suggests insensitivity of failure to the presence of flaws. This is in contrast to failure of monolithic alumina containing a notch of comparable ratio, which would necessarily fail at the notch at a significantly lower peak load compared to that of an un-notched specimen. We observed a reduction in the observed peak load for samples with *(a/w)* ≥ 0.38 and attribute it to greater compliance of those samples, which stems from the fewer intact unit cells at the notch plane where failure occurs. We discovered that failure in these architected meta-materials is governed by the strength of the nodes, and that incipient failure occurs at the nodes with one un-constrained boundary. Once failure is initiated, the crack propagates instantaneously and catastrophically, as expected for a brittle material, along a plane of nodes orthogonal to the loading direction. These results suggest that nanolattices are insensitive to externally introduced defects because their failure mechanism is governed by the stress concentrations within the nodal geometry and by a transition from pure uniaxial tension to bending failure. These findings have significant implications in developing novel materials, which propels architected meta-materials to be particularly lucrative for applications that require simultaneous lightweight, strength, and damage tolerance.

## Additional Information

**How to cite this article**: Montemayor, L. C. *et al.* Insensitivity to Flaws Leads to Damage Tolerance in Brittle Architected Meta-Materials. *Sci. Rep.* **6**, 20570; doi: 10.1038/srep20570 (2016).

## Supplementary Material

Supplementary Information
