# Supplementary material for: Insensitivity to Flaws Leads to Damage Tolerance in Brittle Architected Meta-Materials
Source: Sci Rep. 2016 Feb 3;6:20570. doi: 10.1038/srep20570 (PMC4738344; doi:10.1038/srep20570)
Supplement: Supplementary Information [file srep20570-s1.pdf]

Supplementary Information:

Insensitivity to Flaws Leads to Damage Tolerance in Brittle Architected Meta-Materials

Authors: Montemayor, L. C., Wong, W. H., Zhang, Y. -W., Greer, J. R.

Representative Images of Etched and Non-Etched Samples

The samples are imaged at 5-10kV to non-destructively determine the amount of polymer that has been removed. **Figure 7** shows a representative sample that shows the location of the polymer, which is denoted by a change in contrast of the sample. Samples were etched in O_2 plasma for a total of 16-18 hours, depending on the notch length, and checked for hollowness every 4-6 hours.

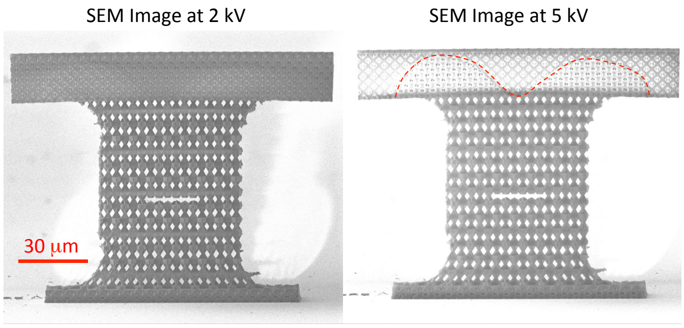

Figure 7: Representative images of polymer etching using different SEM voltages; the red dotted line indicated the etch front of the O_2 plasma in the octet-truss head

Calculation of Stiffness for Grip and Test Gauge Sections

The strength and stiffness of a stretching-dominated cellular solid, such as the octet-truss lattice, scale linearly with the modulus and relative density $\bar{\rho}$, where $\bar{\rho}$ is the ratio between the volume of the lattice unit cell normalized by the total volume occupied by the unit cell[31,48]. These theories assume long, slender, solid bars with rigid nodal connections, though many lightweight foams are made with techniques that produce hollow bars and nodes[43,49,50]. The hollow kagome test sections in this work have a relative density of $\bar{\rho} = 0.02$ and the octet-truss grips have a relative density of $\bar{\rho} = 0.38$. The relative densities are estimated from Solidworks model assuming a constant, perfectly 50nm conformal coating of Al_2O_3 and either hollow (kagome test section) or filled (octet-truss grips) lattice tubes. The SolidWorks model assumes a unit cell size of l =3. 85μm for the kagome test section and l =4.5μm for the octet-truss grips. The dimensions of the voxel, which are used to trace the lattice geometry, have a semi-major axis of a = 1.8 μm and semi-minor axis of b = 0.5 μm. These voxel dimensions were determined by measuring the voxel for a variety of laser power settings for the two-photon lithography process.

The relationship between relative density and stiffness of the hollow kagome lattices was determined using cyclic nano-indentation (G200) in the elastic regime of samples with dimensions of 4x5x3 unit cells in size at a quasi-static strain rate of $\dot{\varepsilon} = 10^{-3}s^{-1}$. The lattice unit cell sizes was l = 3.5μm, the wall thicknesses of the Al_2O_3 coating was 50nm, and the angle of the kagome unit cells was θ = 54.7°. The relative density of the hollow kagome nanolattice was determined to be $\bar{\rho} = 0.02$, similar to those tested in uniaxial tension in this work. Three cycles were used and the modulus is

estimated from the stress-strain loading curve when the displacement does not exceed the elastic limit, as shown in **Figure 8**. The stiffness of the hollow kagome lattice is approximated to be E_{kagome} = 45 MPa.

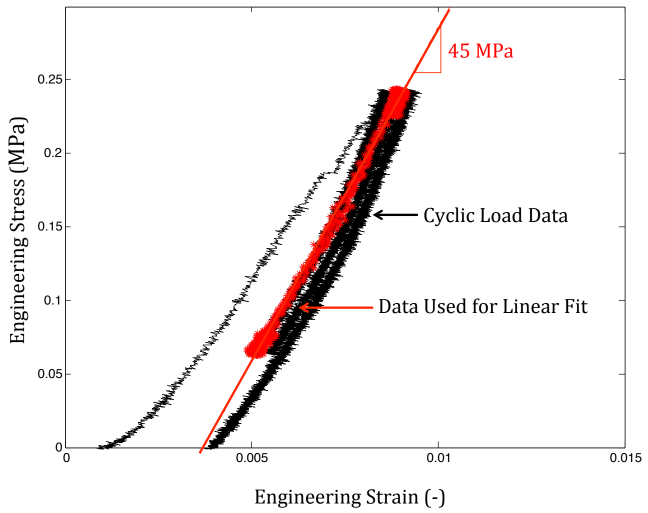

Figure 8: Engineering stress-strain curve for compressive stiffness of 54.7° 3D kagome nanolattices.

The stiffness of the octet-truss grip is calculated using the analytic scaling for a stretching-dominated structure, where $E_{Octet-truss} = 0.3\ E_{bulk}\ \bar{\rho}$ [31,32]. The stiffness E_{bulk} is estimated using the rule-of-mixtures for composites, where

$$E_{bulk} = v_{f,\,polymer}\ E_{polymer} + v_{f,\,Al2O3}\ E_{Al2O3}$$

The volume fractions are calculated using the SolidWorks model and are found to be:

$$v_{total} = 47.3\ \text{um}^3$$
$$v_{polymer} = 38.0\ \text{um}^3$$
$$v_{Al2O3} = 9.31\ \text{um}^3$$

$$v_{f,\,Al2O3} = (v_{total} - v_{polymer})/v_{total} = 0.18$$
$$v_{f,\,polymer} = (v_{total} - v_{Al2O3})/v_{total} = 0.80$$

The bulk material properties of the polymer were found via quasi-static nano-indentation experiments and determined to be $E_{polymer}$ = 2.1 GPa by Meza, et. al.

This gives an estimated E_{bulk} for the composite Al_2O_3/polymer octet-truss bulk material to be

$$E_{bulk} = E_{polymer}\ \ v_{polymer} + E_{Al2O3}\ v_{Al2O3}$$
$$E_{bulk} = 34.2\ \text{GPa}$$

Using the analytic solution for the stiffness of an octet-truss with solid lattice members, the stiffness of the grips is calculated to be:

$$E_{Octet-truss} = 0.3\ E_{bulk}\ \bar{\rho}$$
$$E_{Octet-truss} = 3.90\ GPa$$

Tabulated Results for Various Notch Sizes

(a/w)	***F_{peak} (mN)***	***Number of Samples***
0	2.00 ± 0.19	3
0.23 ± 0.01	1.96 ± 0.20	5
0.32 ± 0.01	2.03 ± 0.17	3
0.37 ± 0.01	1.68 ± 0.09	3
0.44	1.76	1
0.49 ± 0.0	1.70 ± 0.19	2
0.54	1.38	1

Elastic Blunting Phenomena in 3D Hollow Kagome Nanolattices

Fleck et al. predict that the elastic blunting phenomena of the 2D kagome lattice appears in bands of high strain emanating from the notch tip[47]. If elastic blunting occurred for the 3D kagome nanolattice plates in this work, we would expect similar banding behavior emanating from the crack tip, however none is observed, as shown in **Figure 9**.

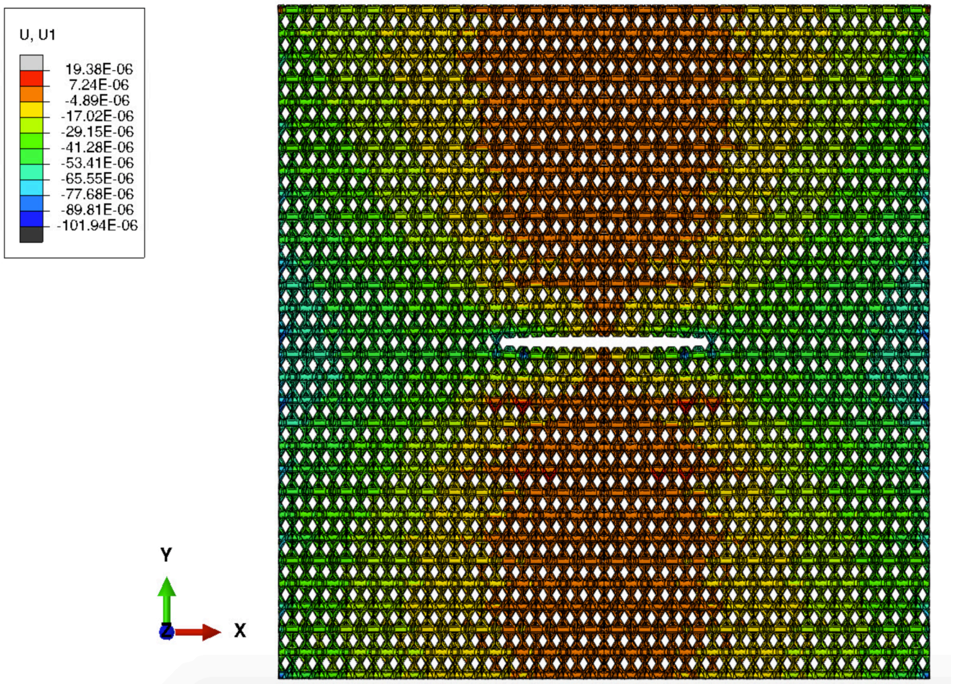

Figure 9: No elastic crack tip blunting is localized near the crack tip for the 3D kagome nanolattice plates; all deformation in the x-direction is localized near the middle of the sample at the moment prior to failure.

Boundary Conditions in FEM Simulation

Hypermesh was used for mesh generation (shown in Figure 10) using IGES generated from Solidworks, and ABAQUS 6.12 for the computations. Computations were carried out on a 16-core, 96 Gb RAM Linux machine, with each analysis executed using 8 cores and requiring approximately 30 hours. The boundary conditions applied to the simulation mimic exactly those applied in the experiment, and are listed below:

- Displacement-controlled boundary conditions at the grips
- Fixed boundary conditions (in all degree-of-freedoms) at the bottom-most of the octet base.
- All other edges are traction-free.

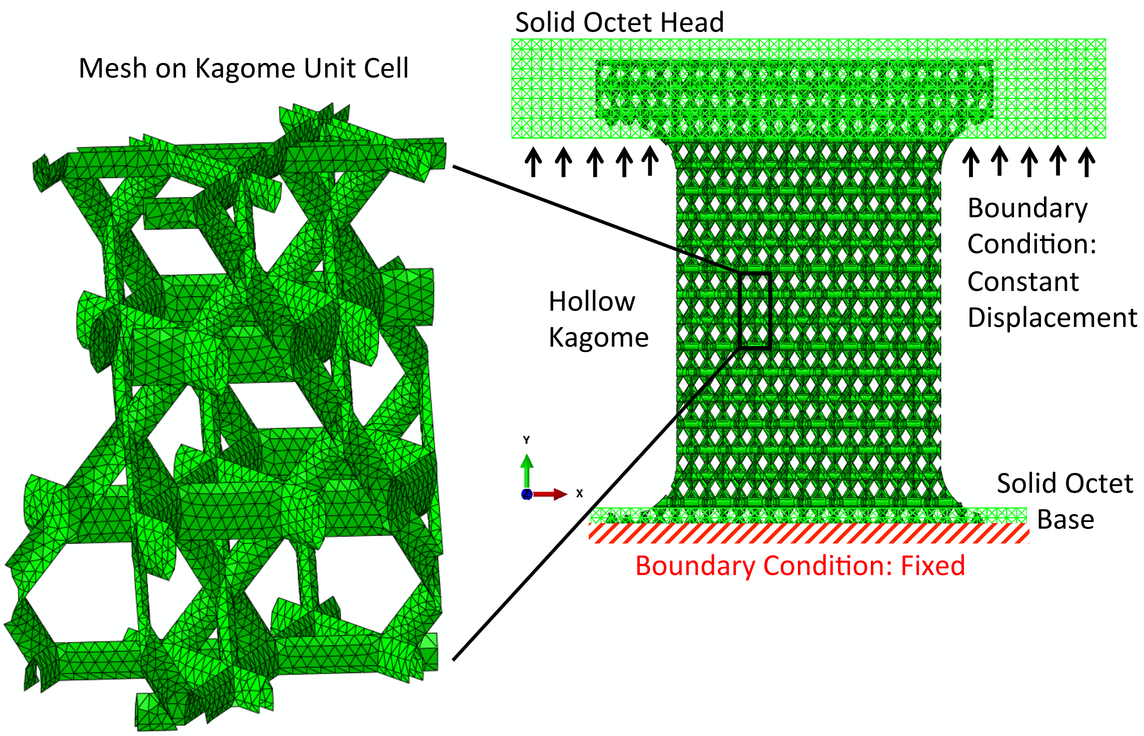

Figure 10 shows the mesh and boundary conditions applied to the nanolattice sample geometry in the FEM simulations.

FEM Model Deformation Showing Bending in Tension Nanolattices Samples

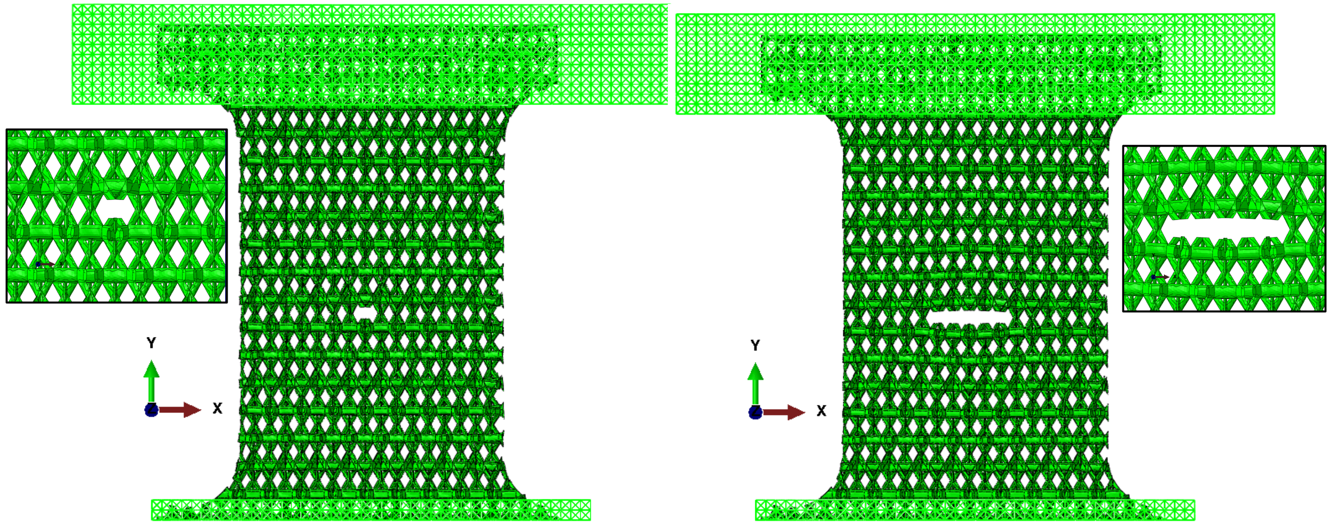

(a) a/w = 0.11 (b) a/w = 0.35

Fig.11 Deformed configurations of samples with (a) a/w = 0.11 and (b) a/w = 0.35, at their respective peak loads. Deformation scale factor of 10 has been applied to aid visualization of the configurations. The respective insets display the close-up views of the deformations in the notches' vicinity.

Figure 11 displays graphically the deformed configurations of samples with a/w = 0.11 and 0.35 at their respective peak loads. The deformations have been magnified 10 times to aid visualization. It can be observed that the sample with a longer notch (a/w = 0.35) experiences more pronounced bending than that of a shorter notch. This is analogous to the case of a cantilever beam pinned at both ends subjected to a mid-span load; for a given amount of load, the deflection (and bending moment) of a longer beam will be larger than that of a shorter one.
